# Cancer Cell-Derived PDGFB Stimulates mTORC1 Activation in Renal Carcinoma

**DOI:** 10.3390/ijms24076447

**Published:** 2023-03-29

**Authors:** Asmaa Y. Abuhamad, Nurul Nadia Mohamad Zamberi, Sakari Vanharanta, Siti Nur Hasanah Mohd Yusuf, M. Aiman Mohtar, Saiful Effendi Syafruddin

**Affiliations:** 1 Bionanotechnology Research Group, Department of Biochemistry, Faculty of Biotechnology and Biomolecular Sciences, Universiti Putra Malaysia, Serdang 43400, Malaysia; 2 UKM Medical Molecular Biology Institute, Universiti Kebangsaan Malaysia, Jalan Yaacob Latiff, Bandar Tun Razak, Kuala Lumpur 56000, Malaysia; 3 Department of Physiology, Faculty of Medicine, University of Helsinki, 00014 Helsinki, Finland; 4 Translational Cancer Medicine Program, Faculty of Medicine, University of Helsinki, 00014 Helsinki, Finland

**Keywords:** renal cancer, mTOR signalling pathway, PDGFB, KLF6, CRISPR/Cas9

## Abstract

Clear cell renal cell carcinoma (ccRCC) is a hypervascular tumor that is characterized by bi-allelic inactivation of the *VHL* tumor suppressor gene and mTOR signalling pathway hyperactivation. The pro-angiogenic factor PDGFB, a transcriptional target of super enhancer-driven KLF6, can activate the mTORC1 signalling pathway in ccRCC. However, the detailed mechanisms of PDGFB-mediated mTORC1 activation in ccRCC have remained elusive. Here, we investigated whether ccRCC cells are able to secrete PDGFB into the extracellular milieu and stimulate mTORC1 signalling activity. We found that ccRCC cells secreted PDGFB extracellularly, and by utilizing KLF6- and PDGFB-engineered ccRCC cells, we showed that the level of PDGFB secretion was positively correlated with the expression of intracellular KLF6 and PDGFB. Moreover, the reintroduction of either KLF6 or PDGFB was able to sustain mTORC1 signalling activity in KLF6-targeted ccRCC cells. We further demonstrated that conditioned media of PDGFB-overexpressing ccRCC cells was able to re-activate mTORC1 activity in KLF6-targeted cells. In conclusion, cancer cell-derived PDGFB can mediate mTORC1 signalling pathway activation in ccRCC, further consolidating the link between the KLF6-PDGFB axis and the mTORC1 signalling pathway activity in ccRCC.

## 1. Introduction

Clear cell renal cell carcinoma (ccRCC) is the most prevalent kidney cancer subtype, accounting for ~75% of all reported cases worldwide [1]. Bi-allelic inactivation of the *VHL* tumor suppressor gene and consequent accumulation of pro-oncogenic hypoxia-inducible factor alpha (HIFα) is the hallmark gatekeeper in ccRCC pathogenesis, contributing to 90% of sporadic ccRCC cases [2,3]. The accumulation of cytoplasmic lipids and glycogens gives rise to the ccRCC distinctive glass-like appearance, which has been attributed to ccRCC metabolic reprogramming [4]. Moreover, ccRCC is highly vascularized due to the upregulation of pro-angiogenic factors [5]. The hyperactivation of mTOR signalling pathway is also frequently observed in ccRCC patients [6]. To this end, inhibitors targeting angiogenesis and the mTOR signalling pathway have been clinically approved as either first-line or second-line treatment against ccRCC [7]. In addition, HIF2α and immune checkpoint inhibitors have also been tested and showed clinical efficacy in some ccRCC patients [8,9]. Nonetheless, these ccRCC monotherapy approaches remain unsatisfactory in terms of patients’ response rate, as well as the rapid development of acquired resistance towards the administered therapy [7]. These could be due to the widespread genetic heterogeneities, inadequate target inhibition, and biological adaptation to an alternative signalling pathway or molecular mechanism that support cells’ survival [2,10]. Therefore, in order to achieve better treatment responses, a number of combinatorial treatments have been tested clinically for their efficacies against ccRCC [11,12].

Cancer cells secrete extracellular proteins, such as growth factors, cytokines, and chemokines that play important roles in supporting cancer pathogenesis [13,14]. These secreted factors modulate growth-associated signalling pathways, reshaping the tumor microenvironment and supporting the formation of the pre-metastatic niche. For instance, squamous skin cancer cells secrete VEGF to stimulate endothelial cell proliferation and subsequent blood vessel formation via paracrine signalling [15]. The secreted VEGF could also promote stemness in an autocrine manner by interacting with the VEGFR co-receptor Nrp1 that resides on the squamous skin cancer cells [15]. Chemokine CXCL1/2, secreted by breast cancer cells, has been shown to promote lung metastasis and chemoresistance by acting on myeloid and endothelial cells via a paracrine signalling cascade [16]. On the other hand, head and neck squamous carcinoma cells enhance their lymph node metastatic capability by secreting chemokine CCL22 that would bind to CCR4 in an autocrine manner [17]. Moreover, cancer cells could evade immune surveillance by secreting factors like PD-L1 that suppresses the activity of PD-1 expressing immune cells [18]. Collectively, cancer secretome has promising clinical values for diagnostic and/or therapeutic purposes owing to their significant functions in promoting tumorigenesis.

PDGF-BB, one of the five members of the PDGF family, is a disulfide-linked homodimeric protein that is formed by two PDGFB subunits. PDGF-BB is a secreted mitogen that plays a role in regulating cells’ proliferation, differentiation, and migration during blood vessel formation and the developmental process [19,20,21]. In addition, PDGF-BB has also been implicated in the pathogenesis of vascular disorders, fibrotic diseases, and cancer [22,23]. In addition to activated platelets, endothelial cells, and fibroblasts, cancer cells have also been reported to secrete PDGF-BB into the extracellular milieu to support their progression and metastatic capability. The secretion of PDGF-BB by liver cancer cells promote hepatic stellate cell proliferation and VEGFA expression that could in turn stimulate angiogenesis [24]. Similarly, glioma cell-derived PDGF-BB enhances angiogenesis by increasing endothelial cell migration and VEGFA expression in these cells [25]. Furthermore, the secretion of PDGF-BB by glioma, melanoma, and fibrosarcoma cells enhance pericyte recruitment to the newly formed blood vessel [25,26,27], where they maintain vascular functions and stability. Additionally, PDGF-BB secreted by cancer cells has been shown to promote lymphangiogenesis and subsequent lymphatic metastasis [28,29].

Super enhancers, or large cluster of enhancers, regulate the expression of genes that are involved in governing key biological processes, such as cell identity and cell fate determination [30]. Moreover, cancer cells are also highly dependent on the functions of genes that are associated with super enhancers [31]. In this regard, a super enhancer-driven transcription factor KLF6 regulates *PDGFB* expression and the transcriptional network that links mTORC1 activity and lipid homeostasis in ccRCC [32]. Although KLF6 was shown to be a putative direct *PDGFB* transcriptional activator in ccRCC, it remains to be elucidated whether ccRCC cells secrete PDGF-BB (hereinafter referred to as PDGFB) and whether KLF6 is involved in modulating this process. Therefore, in this study, we utilized the KLF6- and PDGFB-engineered ccRCC cells to investigate whether ccRCC cells secrete PDGFB extracellularly and are able to stimulate mTORC1 signalling pathway activity. We found that ccRCC cells secreted PDGFB into the extracellular environment, in which the secreted PDGFB level was positively correlated with the intracellular KLF6 and PDGFB expression. Finally, we demonstrated that exogenous expression of either *KLF6* or *PDGFB* and the conditioned media of PDGFB-overexpressing ccRCC cells were able to re-activate mTORC1 signalling in KLF6-targeted ccRCC cells.

## 2. Results

### 2.1. Human ccRCC Cells Secreted PDGFB Extracellularly

**Figure 1:**
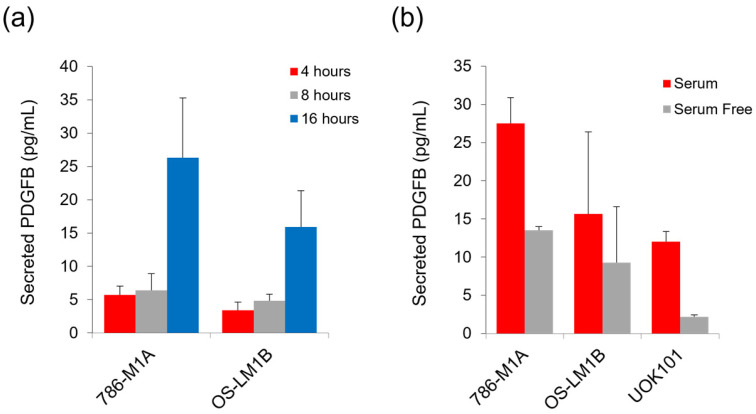
The human ccRCC cells secrete PDGFB into the extracellular environment. (**a**) The level of secreted PDGFB in 786-M1A and OS-LM1B cells’ culture media 4, 8, and 16 h post serum-starvation. (**b**) The comparison between PDGFB secretion level in 786-M1A, OS-LM1B, and UOK101 cells that were either cultured in complete media or serum free media for 16-h. Average of two independent experiments. Error bars represent SD.

To assess whether ccRCC cells secrete PDGFB extracellularly, we first performed ELISA to quantify the level of PDGFB in the serum-starved media that were collected from 786-M1A and OS-LM1B cells at different time points. These cells were cultured in the serum-free media to replicate the condition that we had previously used to study the link between PDGFB and the mTORC1 signalling pathway in ccRCC [32]. We found that the concentration of PDGFB in the collected culture media increased in a time-dependent manner (Figure 1a). Additionally, we also compared the level of secreted PDGFB between ccRCC cells that were cultured in either complete or serum-free media for 16 h. The level of extracellular PDGFB was found to be higher in the ccRCC cells’ complete culture media as compared to serum-free culture media (Figure 1b). Nonetheless, to be consistent with our previous study [32], all of the subsequent experiments were performed in serum-free media. Collectively, these data showed that human ccRCC cells secreted PDGFB into the extracellular milieu.

### 2.2. PDGFB Secretion Level Positively Correlated with Intracellular PDGFB Expression

**Figure 2:**
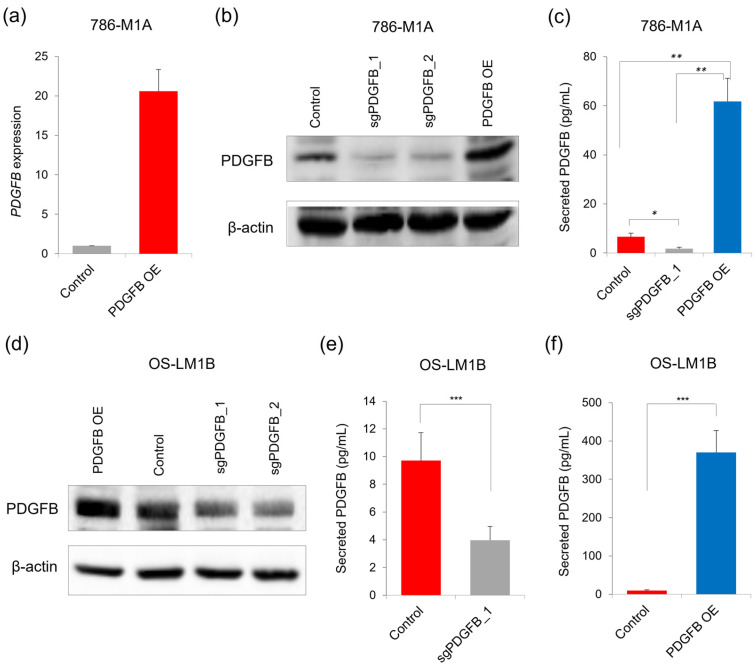
The level of PDGFB secretion by ccRCC cells correlates with intracellular PDGFB expression. (**a**) *PDGFB* mRNA level in the 786-M1A cells that stably expressed the exogenous *PDGFB* CDS. Average of two independent experiments. Error bars represent SD. (**b**) Intracellular PDGFB expression in the PDGFB-targeted and PDGFB-overexpressed 786-M1A cells. (**c**) The level of secreted PDGFB in the PDGFB-targeted and PDGFB-overexpressed 786-M1A cells. Average of three independent experiments. Error bars represent SD. *p*-value by one-way Anova. ** *p* < 0.005, * *p* < 0.05. (**d**) Intracellular PDGFB expression in the PDGFB-targeted and PDGFB-overexpressed OS-LM1B cells. (**e**,**f**) The secreted PDGFB level of the (**e**) PDGFB-targeted and (**f**) PDGFB-overexpressed OS-LM1B cells. Average of three independent experiments. Error bars represent SD. *p*-value by two-tailed *t*-test. *** *p* < 0.0005.

We next assessed whether the modulation of intracellular PDGFB would affect the level of PDGFB secreted by ccRCC cells. To achieve this, we either overexpressed exogenous *PDGFB* or targeted *PDGFB* using CRISPR/Cas9 in the 786-M1A cells. We confirmed PDGFB overexpression in the 786-M1A cells that stably expressed the exogenous *PDGFB* (Figure 2a,b). On the other hand, the *PDGFB* targeting was performed in the pooled 786-M1A cells using two independent sgRNAs, referred to as sgPDGFB_1 and sgPDGFB_2. We observed a reduction in the expression of intracellular PDGFB in the 786-M1A cells that were transduced with the sgPDGFB constructs as compared to the control cells (transduced with non-targeting sgRNA construct) (Figure 2b). We then collected the respective serum-starved media from these PDGFB-engineered and control 786-M1A cells and subjected the media to PDGFB ELISA. Relative to the control cells, we found that the PDGFB-targeted 786-M1A cells had the lowest level of secreted PDGFB, whereas the PDGFB overexpressing cells secreted more PDGFB into the extracellular environment (Figure 2c).

To consolidate these observations, we also modulated the expression of intracellular PDGFB in the OS-LM1B cells similar to those established for 786-M1A cells. We confirmed that they were increased and decreased in intracellular PDGFB expression in the PDGFB-overexpressing and PDGFB-targeted OS-LM1B cells, respectively (Figure 2d). We found that the OS-LM1B cells that had the PDGFB targeted using CRISPR/Cas9 secreted less PDGFB extracellularly compared to the OS-LM1B control cells (Figure 2e). On the other hand, a high level of extracellular PDGFB was detected in the PDGFB-overexpressing OS-LM1B cells’ culture media (Figure 2f). Overall, it can be inferred from these findings that ccRCC cells indeed secreted PDGFB into the extracellular milieu, and the secretion level positively correlated with the intracellular PDGFB expression.

### 2.3. KLF6 Regulated PDGFB Expression and Secretion in ccRCC Cells

**Figure 3:**
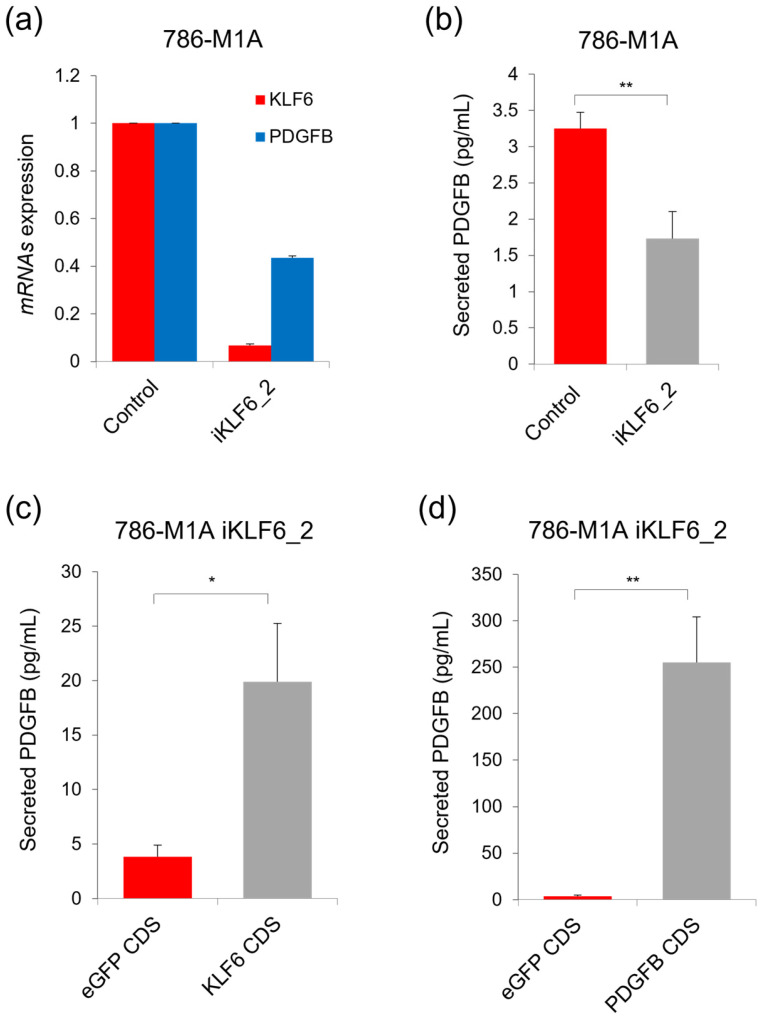
PDGFB secretion level in 786-M1A cells correlate with the expression of its upstream regulator KLF6. (**a**) The expression of KLF6 and PDGFB in the KLF6-repressed 786-M1A cells. Average of three independent experiments. Error bars represent SD. (**b**) The level of secreted PDGFB of the KLF6-repressed 786-M1A cells. (**c**) The secreted PDGFB level upon the reintroduction of exogenous KLF6 CDS into the KLF6-repressed 786-M1A cells. (**d**) PDGFB secretion level in the KLF6-repressed 786-M1A cells reintroduced with exogenous PDGFB CDS. (**b**–**d**) Average of three independent experiments. Error bars represent SD. *p*-value by two-tailed *t*-test. ** *p* < 0.005, * *p* < 0.05.

We have previously reported that *PDGFB* expression in ccRCC was directly transactivated by transcription factor KLF6 [32]. Therefore, we were prompted to measure the level of extracellular PDGFB in the previously generated 786-M1A iKLF6_2 cells, which have KLF6 stably repressed using CRISPRi approach [32]. We confirmed that there were strong *KLF6* repression and consequent *PDGFB* downregulation in these cells (Figure 3a). We found that the 786-M1A iKLF6_2 cells secreted a lower amount of PDGFB compared to the 786-M1A control cells (Figure 3b), which correlated with the reduced expression of intracellular *PDGFB* in these KLF6-repressed cells. We next measured the level of secreted PDGFB in the 786-M1A iKLF6_2 cells that expressed either exogenous *KLF6* CDS or *PDGFB* CDS. *KLF6* overexpression and subsequent *PDGFB* upregulation were confirmed in the 786-M1A iKLF6_2 cells that stably expressed the exogenous *KLF6* CDS (Supplementary Figure S1a,b). Consistent with the upregulation of intracellular *PDGFB*, we detected a significant increase in PDGFB level in the culture media collected from these *KLF6* CDS-expressing 786-M1A iKLF6_2 cells (Figure 3c). Moreover, the reintroduction of *PDGFB* CDS into these 786-M1A iKLF6_2 cells profoundly increased the level of PDGFB that got secreted into the extracellular environment (Supplementary Figure S1c and Figure 3d).

**Figure 4:**
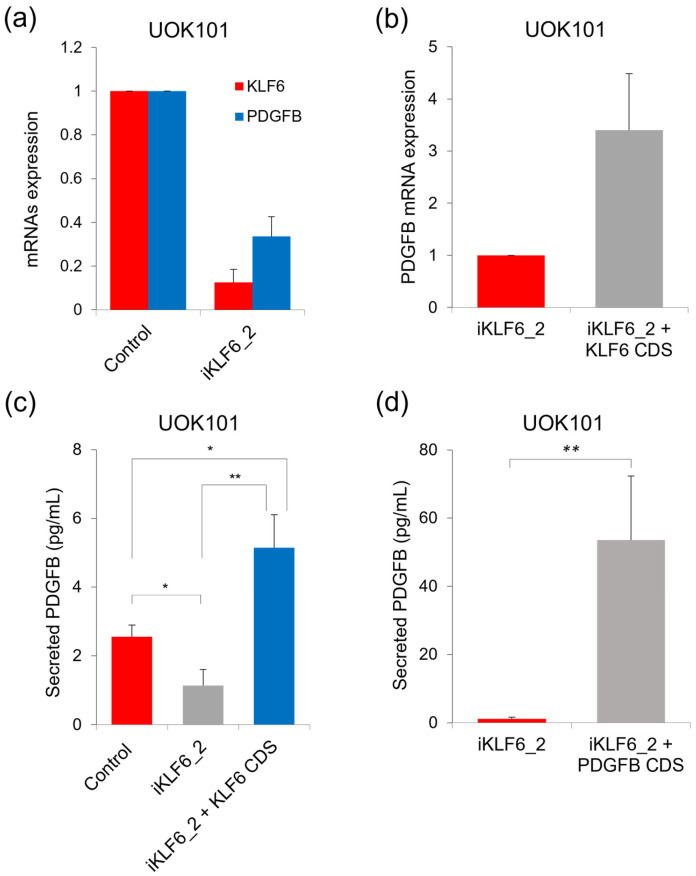
KLF6 modulates PDGFB expression and secretion in UOK101 cells. (**a**) The expression of *KLF6* and *PDGFB* in the KLF6-repressed UOK101 cells. (**b**) The expression of *PDGFB* in the KLF6-repressed UOK101 cells that expressed exogenous *KLF6* CDS. Average of two independent experiments. Error bars represent SD. (**c**) PDGFB secretion level in the UOK101 control cells, KLF6-repressed UOK101 cells, and KLF6-repressed UOK101 cells that were reintroduced with exogenous *KLF6* CDS. Average of three independent experiments. Error bars represent SD. *p*-value by one-way Anova. ** *p* < 0.005, * *p* < 0.05. (**d**) The secreted PDGFB level upon the reintroduction of exogenous *PDGFB* CDS into the KLF6-repressed UOK101 cells. Average of three independent experiments. Error bars represent SD. *p*-value by two-tailed *t*-test. ** *p* < 0.005.

We further validated the findings in Figure 3 by measuring the level of extracellular PDGFB in an additional set of KLF6-modulated ccRCC cells, which were the UOK101 cells. CRISPRi-mediated KLF6 repression in UOK101 cells reduced *PDGFB* expression, whereby the reintroduction of exogenous *KLF6* CDS in these cells resulted in *PDGFB* upregulation (Figure 4a,b and Supplementary Figure S2a). We found that the PDGFB secretion level in these UOK101 cells also positively correlated with the expression of intracellular *PDGFB* that was regulated by the transcription factor KLF6 (Figure 4c). As expected, the KLF6-repressed UOK101 cells that stably expressed exogenous *PDGFB* CDS secreted a high level of PDGFB extracellularly (Figure 4d and Supplementary Figure S2b). Collectively, these data clearly demonstrated that ccRCC cells secreted PDGFB into the extracellular environment and that the secretion level was positively correlated with the expression of intracellular PDGFB and its upstream regulator KLF6. Importantly, we have corroborated the functional link between one of the strongest super enhancers and the regulation of PDGFB expression and secretion in ccRCC.

### 2.4. Secreted PDGFB Stimulates mTORC1 Signalling Pathway Activation

**Figure 5:**
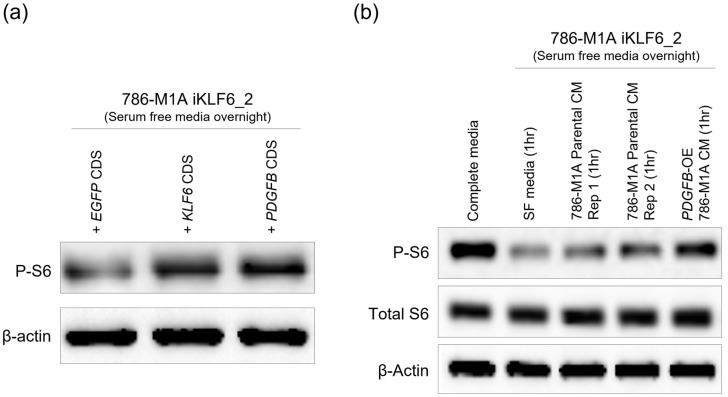
Secreted PDGFB stimulates mTORC1 signalling pathway activation. (**a**) Phosphorylated S6 Western blot of 786-M1A iKLF6_2 that were reintroduced with *eGFP* CDS, *KLF6* CDS or *PDGFB* CDS. Representative of two experiments. (**b**) mTORC1 activity of the 786-M1A iKLF6_2 cells that were cultured with serum free media (SF), conditioned media (CM) of 786-M1A parental or PDGFB-overexpressing 786-M1A cells for 1 h following overnight serum starvation. Representative of two experiments.

We have previously demonstrated the role of the KLF6-PDGFB axis in modulating mTORC1 activity in ccRCC. Repressing either KLF6 or PDGFB impaired mTORC1 activity, whereas supplementing the KLF6-repressed cells with recombinant human PDGFB re-activated the mTORC1 signalling pathway in ccRCC [32]. However, whether cancer cell-derived PDGFB is also able to activate mTORC1 activity in KLF6-depleted cells has remained unclear. Since the reintroduction of exogenous *KLF6* and *PDGFB* increased the PDGFB secretion level of the KLF6-repressed cells (Figure 3c,d and Figure 4c,d), we postulated that these cells would have sustained mTORC1 activity. To assess this, we blotted for phosphorylated S6 protein, a read-out for mTORC1 activity, in the KLF6- and PDGFB-expressing 786-M1A iKLF6_2 cells that underwent serum starvation overnight. In line with our hypothesis, we found that the KLF6- and PDGFB-expressing cells had more mTORC1 signalling activity compared to the 786-M1A iKLF6_2 control cells (Figure 5a).

We next tested whether the secreted PDGFB was able to stimulate mTORC1 activity in a paracrine manner. The mTORC1-impaired 786-M1A iKLF6_2 cells were serum-starved overnight and cultured for an hour on the following day with (i) serum-free media, (ii) 786-M1A parental cells’, or (iii) PDGFB-overexpressing 786-M1A cells’ conditioned media. Prior to collecting the conditioned media, these parental and PDGFB-overexpressing 786-M1A cells were cultured in serum-free media overnight. Supplementing the KLF6-repressed cells with the conditioned media from PDGFB-overexpressing cells resulted in the reactivation of the mTORC1 activity (Figure 5b). On the other hand, the conditioned media from the 786-M1A parental cells was also able to stimulate the mTORC1 activity, although the magnitude of induction was much lower than the conditioned media of PDGFB-overexpressing cells (Figure 5b). To complement these results, we replicated these experiments using the UOK101 iKLF6_2 cells. Consistent with our previous and present findings, we observed a reduced mTORC1 activity in the KLF6-repressed UOK101 cells. Furthermore, the reintroduction of exogenous *PDGFB* CDS or culturing these UOK101 iKLF6_2 cells with conditioned media from PDGFB-overexpressing cells were able to sustain and reactivate the mTORC1 signalling pathway, respectively (Supplementary Figure S3).

## 3. Discussion

By utilizing KLF6- and PDGFB-engineered ccRCC cells, we confirmed that ccRCC cells secrete PDGFB into the extracellular space. We found that PDGFB targeting reduced the level of extracellular PDGFB, whereas the overexpression of PDGFB increased the PDGFB secretion level. This was in line with the fact that the level of most, if not all, secreted proteins would depend on the expression of their intracellular counterparts. Altering the expression of these intracellular proteins, either by genetic or chemical means, would affect the secretion level. Intriguingly, we found that the PDGFB secretion level was also correlated with the expression of its upstream transcriptional activator KLF6. We have previously reported that KLF6 was highly expressed in ccRCC, and its expression was driven by one of the strongest and robust super enhancers in ccRCC [32]. Based on these findings, we postulated that the high expression of KLF6 enhances *PDGFB* transcriptional activation that could in turn play an important role in supporting ccRCC pathogenesis. Whilst targeting KLF6 impaired ccRCC cell growth and lung colonization capabilities [32], it is worthwhile to test the effect of direct PDGFB perturbation on ccRCC cell phenotypes in a future study. Our present findings corroborated the functional link between the KLF6 super enhancer locus and the transcriptional regulation of the angiogenesis-promoting PDGFB in ccRCC. This is in line with other reported roles of super enhancers as drivers of the expression of genes that regulate and maintain cancer phenotypes [33,34].

One of ccRCC hallmark features observed in patients is the frequent hyperactivation of the mTOR signalling pathway [6,35]. Following the report that PDGFB is one of the mTOR signalling pathway agonists [36], we previously showed that supplementing the KLF6-targeted ccRCC cells with recombinant human PDGFB re-activated the impaired mTORC1 activity in these cells [32]. The secretion of PDGFB by ccRCC cells and the ability of conditioned ccRCC media to activate mTORC1 signalling in KLF6 depleted cells, as reported herein, consolidates our previous findings. It is noteworthy to highlight that our results unraveled a molecular connection between pro-angiogenic factors and mTOR pathway activation, which are the two approved therapeutic targets in ccRCC [7]. Concordantly, a combinatorial treatment of lenvatinib (a multi RTKs inhibitor including PDGFR) and mTOR inhibitor everolimus on metastatic RCC patients who have progressed after one previous VEGF-targeted therapy increased the progression-free survival over either drug alone [37]. In a separate trial on untreated advanced RCC patients, a combination of lenvatinib with either pembrolizumab (PD-L1 monoclonal antibody) or everolimus prolonged the progression-free survival over sunitinib treatment alone [38]. The secretion of PDGFB by ccRCC cells reported herein could prompt the idea of the potential development and use of monoclonal antibodies against secreted PDGFB in treating ccRCC. There have been reports examining the correlation between intracellular PDGFB/PDGFRβ expression and RCC stages or prognosis [39,40]. However, to our knowledge, there is no study directly evaluating the serum PDGFB level in RCC in comparison to a healthy control and across different tumor stages. This knowledge is crucial to determine the possibility of not only targeting the circulating PDGFB, but also for the development of diagnostic and/or prognostic biomarkers based on the circulating PDGFB level.

**Figure 6:**
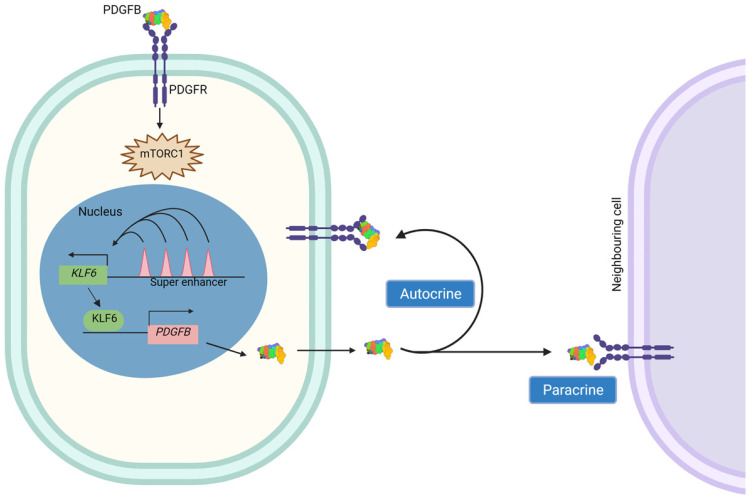
Summary—The mode of mTORC1 signaling pathway stimulation in ccRCC by cancer-derived PDGFB.

In conclusion, secretion of PDGFB by ccRCC cells is able to induce mTORC1 activity in the neighboring ccRCC cells in a paracrine manner. In this regard, the secreted PDGFB could also possibly act on the same cells in an autocrine manner (Figure 6). Our findings further establish the role of one of the strongest super enhancers in ccRCC as a modulator of mTORC1 activation via the KLF6-PDGFB transcriptional axis. Thus, the common hyperactivation of the mTORC1 signalling pathway in ccRCC could, at least in part, be a consequence of the high expression of KLF6 and the downstream transcription of the mTOR agonist *PDGFB*.

## 4. Materials and Methods

### 4.1. Cell Lines and Reagents

The human ccRCC cell lines used in this study were 786-M1A, OS-LM1, UOK101, A498, and 769-P. The 786-M1A and OS-LM1 cell lines were obtained from J. Massagué (Memorial Sloan Kettering Cancer Center, New York, NY, USA). The 786-M1A and OS-LM1 are the metastatic derivative of 786-O and OS-RC2 cells, respectively, which were established and described previously [41]. The UOK101 cell line was obtained from Marston Linehan (National Cancer Institute, Bethesda, MD, USA). The A498 and 769-P cell lines were obtained from the American Type Culture Collection (ATCC, Manassas, VA, USA). These cell lines were confirmed to be mycoplasma negative using the e-MycoTM Mycoplasma PCR Detection Kit (Intron, Kirkland, WA, USA). All of these human ccRCC cell lines were maintained in RPMI-1640 medium (Nacalai Tesque, Kyoto, Japan), supplemented with 10% FBS (Tico Europe, Amstelveen, The Netherlands) and 1% penicillin-streptomycin solution (Nacalai Tesque). The HEK293T cells used for lentivirus production were cultured in DMEM (Nacalai Tesque), supplemented with 10% FBS and 1% penicillin-streptomycin solution. Puromycin and Hygromycin B solution were purchased from Invivogen and Nacalai Tesque, respectively.

The constitutive Cas9 plasmid, LentiCas9-Blast, was a gift from Feng Zhang (Addgene plasmid #52962) [42]. The sgRNA expression plasmid, pKLV-U6-gRNA(BbsI)-PGKHygro2AeGFP, was previously generated [32]. This plasmid was modified from pKLV-U6-gRNA(BbsI)-PGKpuro2ABFP, which was a gift from Kosuke Yusa (Addgene plasmid #50946) [43]. The pLVX-Puro plasmid (Clontech #632164) was used to express the exogenous PDGFB coding sequence (CDS). The lentivirus packaging plasmids psPAX2 (Addgene plasmid #12260) and pMD2.G (Addgene plasmid #122259) were gifts from Didier Trono. All primers and sgRNA constructs used in this study were purchased for Integrated DNA Technologies (IDT). These sequences are listed in Supplementary Table S1.

### 4.2. sgPDGFB Cloning and Bacteria Transformation

The complementary sgPDGFB top and bottom strands were purchased separately. These strands were designed to harbor *BbsI* restriction overhangs at their respective 5′ and 3′ ends for ligation into *BbsI*-digested pKLV-U6-gRNA(BbsI)-PGKHygro2AeGFP plasmid. The top and bottom strands were annealed and subjected to 5′ end phosphorylation using T4 polynucleotide kinase (New England Biolabs, Ipswich, MA, USA). Ligation was performed using T4 Ligase (New England Biolabs) at 16 °C overnight and the ligated plasmid was transformed into the chemically competent DH5α *E. coli* strain (New England Biolabs). The presence of sgPDGFB construct within the expression plasmid was verified via Sanger sequencing.

### 4.3. Plasmid and Total RNA Extraction

Plasmid was extracted from the transformed bacteria culture using Monarch Plasmid Miniprep Kit (New England Biolabs) according to the manufacturer’s protocols. Total RNA was extracted from the cells using Trizol^TM^ reagent (Thermo Fisher, Waltham, MA, USA) by following the manufacturer’s protocols. The yield and purity of the extracted plasmids and total RNA were determined using the NanoDrop^TM^ 2000c Spectrophotometer (Thermo Fisher).

### 4.4. Lentivirus Transduction

The HEK293T cells were co-transfected with the mixture of lentivirus packaging plasmids and plasmid of interest using Attractene transfection reagent (Qiagen, Hilden, Germany) according to the manufacturer’s recommendations. Media containing the lentivirus was collected 72 h post-transfection and filtered through MinisartNML 0.45 μM syringe filter (Sartorius, Göttingen, Germany). For lentiviral transduction, the filtered media containing the lentivirus was added onto the cells, which were at 60–70% confluency, in the presence of 8 μg/mL Polybrene (Millipore, Burlington, MA, USA).

### 4.5. cDNA Synthesis and qRT-PCR

Total RNA was converted into cDNA using the LunaScript RT SuperMix Kit (New England Biolabs) according to the manufacturer’s recommendations. The qRT-PCR was performed using the Luna Universal Probe qPCR Master Mix (New England Biolabs) and 20× pre-designed TaqMan gene expression probes (Thermo Fisher) on the CFX96 Touch Real-Time PCR Detection System (Bio-Rad, Hercules, CA, USA) according to the manufacturer’s recommendations. The following TaqMan probes were used: KLF6 (Hs00810569_m1), PDGFB (Hs00966522_m1), and TBP (Hs00427620_m1). The Ct values of the gene of interest were normalized using the Ct value of the housekeeping control, TBP. The gene expression fold change between the samples was calculated using the 2^−ΔΔCt^ method.

### 4.6. Protein Extraction and Western Blot

Cells were either trypsinized or scraped, followed by cell lysis on ice using 1× RIPA lysis buffer containing 1:100 protease inhibitor cocktail (Nacalai Tesque) and 1:100 phosphatase inhibitor cocktail (Thermo Fisher). The Pierce BCA Protein Assay Kit (Thermo Fisher) was used to determine the protein lysate concentration according to the manufacturer’s protocols. Equal amount of protein samples were boiled in 1× Trident Laemmli SDS Sample Buffer (GeneTex, Irvine, CA, USA) containing 8% Beta-mercaptoethanol. The protein samples were separated in 10% SDS-PAGE gel and transferred onto nitrocellulose membrane (GE Healthcare Amersham, (GE Healthcare, Chicago, IL, USA; Amersham, UK). The membrane was blocked with 5% non-fat dry milk in 0.1% TBS-Tween and blotted with primary antibody overnight at 4 °C. Secondary antibody was added onto the membrane on the following day and incubated for an hour at room temperature. Signals were developed using Pierce™ ECL Western Blotting Substrate (Thermo Fisher) and visualized using the ChemiDoc XRS+ Gel Imaging System (Bio-Rad). For mTORC1 activity assessment, the cells were serum starved overnight and subjected to Western blotting as described above. Instead of non-fat dry milk, 5% BSA was used for blocking the membrane and diluting the antibodies. Primary antibodies used were PDGFB (Santa Cruz, Dallas, TX, USA, sc-365805, 1:1000), P-S6 ribosomal (Cell Signaling Technology, Danvers, MA, USA, Ser235/236, #4857, 1:3000), S6 ribosomal (Cell Signaling Technology, #2317, 1:1000), and B-actin (Santa Cruz, sc-69879, 1:5000). Secondary antibodies were polyclonal rabbit anti-mouse IgG/HRP (Dako, Santa Clara, CA, USA, P0260, 1:1000) and polyclonal swine anti-rabbit IgG/HRP conjugated (Dako, P0217, 1:1000). 

### 4.7. Exogenous PDGFB Expression

The PDGFB CDS was amplified from the cDNA of 786-M1A cells using the Accuprime Pfx Supermix (Thermo Fisher). The amplified PDGFB CDS harbored the *EcoRI* and *XbaI* restriction sites upstream of the start codon and downstream of the stop codon, respectively, for ligation into the pLVX-Puro plasmid. The ligation and bacteria transformation were performed according to the cloning strategy described in the previous subsection. The presence of the ligated PDGFB CDS within the expression plasmid was confirmed via Sanger sequencing.

### 4.8. Acetone Protein Precipitation

The cells were washed twice with 1× PBS and cultured in serum free media overnight. The media was collected and spun down at 1000 RPM for 5 min to pellet the cells and debris. The media was transferred into conical tube, and 4 volume of ice-cold acetone was added and mixed well. The mixture was incubated at −20 °C overnight and spun down at 13,000× *g* for 10 min at 4 °C on the following day. The supernatant was removed, and the precipitated proteins were dissolved in 1× RIPA lysis buffer containing 1:100 protease inhibitor cocktails. The dissolved proteins were subjected to PDGFB Western blot.

### 4.9. PDGF-BB ELISA

The cells were washed twice with 1× PBS and cultured in serum free media overnight. On the next day, the media was collected and spun down at 1000 RPM for 5 min to pellet the cells and debris. The media were then subjected to PDGFB ELISA using RayBio Human PDGF-BB ELISA Kit (RayBiotech, Peachtree Corners, GA, USA) according to manufacturer’s recommendations. The absorbance of standards and samples were read at 450 nm using the Varioskan LUX Multimode Microplate Reader (Thermo Fisher). The concentration of extracellular PDGFB in the media was determined using the Four Parameter Logistic Curve (https://www.myassays.com/four-parameter-logistic-curve.assay (accessed on 15 April 2022)).

### 4.10. Statistical Analysis

Two-tailed unpaired *t*-test or one-way ANOVA were used for the PDGF-BB ELISA experiments, and *p*-values lower than 0.05 were considered statistically significant. For the qRT-PCR, three independent experiments are shown unless stated otherwise in the figure legend. Each of the experiment is the average of three technical replicates.

## Data Availability

Not applicable.

## Supplementary Materials

The following supporting information can be downloaded at: https://www.mdpi.com/article/10.3390/ijms24076447/s1.

## References

[1] Hsieh J.J., Purdue M.P., Signoretti S., Swanton C., Albiges L., Schmidinger M., Heng D.Y., Larkin J., Ficarra V. (2017). Renal Cell Carcinoma. Nat. Rev. Dis. Primers.

[2] Turajlic S., Xu H., Litchfield K., Rowan A., Horswell S., Chambers T., O’Brien T., Lopez J.I., Watkins T.B.K., Nicol D. (2018). Deterministic Evolutionary Trajectories Influence Primary Tumor Growth: TRACERx Renal. Cell.

[3] Shen C., Kaelin W.G. (2013). The VHL/HIF Axis in Clear Cell Renal Carcinoma. Semin. Cancer Biol..

[4] Wettersten H.I., Aboud O.A., Lara P.N., Weiss R.H. (2017). Metabolic Reprogramming in Clear Cell Renal Cell Carcinoma. Nat. Rev. Nephrol..

[5] Qian C.-N., Huang D., Wondergem B., Teh B.T. (2009). Complexity of Tumor Vasculature in Clear Cell Renal Cell Carcinoma. Cancer.

[6] Pantuck A.J., Seligson D.B., Klatte T., Yu H., Leppert J.T., Moore L., O’Toole T., Gibbons J., Belldegrun A.S., Figlin R.A. (2007). Prognostic Relevance of the MTOR Pathway in Renal Cell Carcinoma: Implications for Molecular Patient Selection for Targeted Therapy. Cancer.

[7] Choueiri T.K., Motzer R.J. (2017). Systemic Therapy for Metastatic Renal-Cell Carcinoma. N. Engl. J. Med..

[8] Choueiri T.K., Bauer T.M., Papadopoulos K.P., Plimack E.R., Merchan J.R., McDermott D.F., Michaelson M.D., Appleman L.J., Thamake S., Perini R.F. (2021). Inhibition of Hypoxia-Inducible Factor-2α in Renal Cell Carcinoma with Belzutifan: A Phase 1 Trial and Biomarker Analysis. Nat. Med..

[9] Motzer R.J., Escudier B., McDermott D.F., George S., Hammers H.J., Srinivas S., Tykodi S.S., Sosman J.A., Procopio G., Plimack E.R. (2015). Nivolumab versus Everolimus in Advanced Renal-Cell Carcinoma. N. Engl. J. Med..

[10] Rini B.I., Atkins M.B. (2009). Resistance to Targeted Therapy in Renal-Cell Carcinoma. Lancet Oncol..

[11] Choueiri T.K., Powles T., Burotto M., Escudier B., Bourlon M.T., Zurawski B., Oyervides Juárez V.M., Hsieh J.J., Basso U., Shah A.Y. (2021). Nivolumab plus Cabozantinib versus Sunitinib for Advanced Renal-Cell Carcinoma. N. Engl. J. Med..

[12] Kammerer-Jacquet S.-F., Deleuze A., Saout J., Mathieu R., Laguerre B., Verhoest G., Dugay F., Belaud-Rotureau M.-A., Bensalah K., Rioux-Leclercq N. (2019). Targeting the PD-1/PD-L1 Pathway in Renal Cell Carcinoma. Int. J. Mol. Sci..

[13] Karagiannis G.S., Pavlou M.P., Diamandis E.P. (2010). Cancer Secretomics Reveal Pathophysiological Pathways in Cancer Molecular Oncology. Mol. Oncol..

[14] Madden E.C., Gorman A.M., Logue S.E., Samali A. (2020). Tumour Cell Secretome in Chemoresistance and Tumour Recurrence. Trends Cancer.

[15] Beck B., Driessens G., Goossens S., Youssef K.K., Kuchnio A., Caauwe A., Sotiropoulou P.A., Loges S., Lapouge G., Candi A. (2011). A Vascular Niche and a VEGF–Nrp1 Loop Regulate the Initiation and Stemness of Skin Tumours. Nature.

[16] Acharyya S., Oskarsson T., Vanharanta S., Malladi S., Kim J., Morris P.G., Manova-Todorova K., Leversha M., Hogg N., Seshan V.E. (2012). A CXCL1 Paracrine Network Links Cancer Chemoresistance and Metastasis. Cell.

[17] Tsujikawa T., Yaguchi T., Ohmura G., Ohta S., Kobayashi A., Kawamura N., Fujita T., Nakano H., Shimada T., Takahashi T. (2013). Autocrine and Paracrine Loops between Cancer Cells and Macrophages Promote Lymph Node Metastasis via CCR4/CCL22 in Head and Neck Squamous Cell Carcinoma. Int. J. Cancer.

[18] Cha J.-H., Chan L.-C., Li C.-W., Hsu J.L., Hung M.-C. (2019). Mechanisms Controlling PD-L1 Expression in Cancer. Mol. Cell.

[19] Bjarnegård M., Enge M., Norlin J., Gustafsdottir S., Fredriksson S., Abramsson A., Takemoto M., Gustafsson E., Fässler R., Betsholtz C. (2004). Endothelium-Specific Ablation of PDGFB Leads to Pericyte Loss and Glomerular, Cardiac and Placental Abnormalities. Development.

[20] Xie H., Cui Z., Wang L., Xia Z., Hu Y., Xian L., Li C., Xie L., Crane J., Wan M. (2014). PDGF-BB Secreted by Preosteoclasts Induces Angiogenesis during Coupling with Osteogenesis. Nat. Med..

[21] Munk A.S., Wang W., Bèchet N.B., Eltanahy A.M., Cheng A.X., Sigurdsson B., Benraiss A., Mäe M.A., Kress B.T., Kelley D.H. (2019). PDGF-B Is Required for Development of the Glymphatic System. Cell Rep..

[22] Andrae J., Gallini R., Betsholtz C. (2008). Role of Platelet-Derived Growth Factors in Physiology and Medicine. Genes Dev..

[23] Heldin C.-H., Lennartsson J., Westermark B. (2018). Involvement of Platelet-Derived Growth Factor Ligands and Receptors in Tumorigenesis. J. Intern. Med..

[24] Lu Y., Lin N., Chen Z., Xu R. (2015). Hypoxia-Induced Secretion of Platelet-Derived Growth Factor-BB by Hepatocellular Carcinoma Cells Increases Activated Hepatic Stellate Cell Proliferation, Migration and Expression of Vascular Endothelial Growth Factor-A. Mol. Med. Rep..

[25] Guo P., Hu B., Gu W., Xu L., Wang D., Huang H.-J.S., Cavenee W.K., Cheng S.-Y. (2003). Platelet-Derived Growth Factor-B Enhances Glioma Angiogenesis by Stimulating Vascular Endothelial Growth Factor Expression in Tumor Endothelia and by Promoting Pericyte Recruitment. Am. J. Pathol..

[26] Furuhashi M., Sjöblom T., Abramsson A., Ellingsen J., Micke P., Li H., Bergsten-Folestad E., Eriksson U., Heuchel R., Betsholtz C. (2004). Platelet-Derived Growth Factor Production by B16 Melanoma Cells Leads to Increased Pericyte Abundance in Tumors and an Associated Increase in Tumor Growth Rate. Cancer Res..

[27] Abramsson A., Lindblom P., Betsholtz C. (2003). Endothelial and Nonendothelial Sources of PDGF-B Regulate Pericyte Recruitment and Influence Vascular Pattern Formation in Tumors. J. Clin. Investig..

[28] Cao R., Björndahl M.A., Religa P., Clasper S., Garvin S., Galter D., Meister B., Ikomi F., Tritsaris K., Dissing S. (2004). PDGF-BB Induces Intratumoral Lymphangiogenesis and Promotes Lymphatic Metastasis. Cancer Cell.

[29] Kodama M., Kitadai Y., Sumida T., Ohnishi M., Ohara E., Tanaka M., Shinagawa K., Tanaka S., Yasui W., Chayama K. (2010). Expression of Platelet-Derived Growth Factor (PDGF)-B and PDGF-Receptor β Is Associated with Lymphatic Metastasis in Human Gastric Carcinoma. Cancer Sci..

[30] Hnisz D., Abraham B.J., Lee T.I., Lau A., Saint-André V., Sigova A.A., Hoke H.A., Young R.A. (2013). Super-Enhancers in the Control of Cell Identity and Disease. Cell.

[31] Tang F., Yang Z., Tan Y., Li Y. (2020). Super-Enhancer Function and Its Application in Cancer Targeted Therapy. NPJ Precis. Oncol..

[32] Syafruddin S.E., Rodrigues P., Vojtasova E., Patel S.A., Zaini M.N., Burge J., Warren A.Y., Stewart G.D., Eisen T., Bihary D. (2019). A KLF6-Driven Transcriptional Network Links Lipid Homeostasis and Tumour Growth in Renal Carcinoma. Nat. Commun..

[33] Lovén J., Hoke H.A., Lin C.Y., Lau A., Orlando D.A., Vakoc C.R., Bradner J.E., Lee T.I., Young R.A. (2013). Selective Inhibition of Tumor Oncogenes by Disruption of Super-Enhancers. Cell.

[34] Sengupta S., George R.E. (2017). Super-Enhancer-Driven Transcriptional Dependencies in Cancer. Trends Cancer.

[35] Robb V.A., Karbowniczek M., Klein-Szanto A.J., Henske E.P. (2007). Activation of the MTOR Signaling Pathway in Renal Clear Cell Carcinoma. J. Urol..

[36] Razmara M., Heldin C.-H., Lennartsson J. (2013). Platelet-Derived Growth Factor-Induced Akt Phosphorylation Requires MTOR/Rictor and Phospholipase C-Γ1, Whereas S6 Phosphorylation Depends on MTOR/Raptor and Phospholipase D. Cell Commun. Signal..

[37] Motzer R.J., Hutson T.E., Glen H., Michaelson M.D., Molina A., Eisen T., Jassem J., Zolnierek J., Maroto J.P., Mellado B. (2015). Lenvatinib, Everolimus, and the Combination in Patients with Metastatic Renal Cell Carcinoma: A Randomised, Phase 2, Open-Label, Multicentre Trial. Lancet Oncol..

[38] Motzer R., Alekseev B., Rha S.-Y., Porta C., Eto M., Powles T., Grünwald V., Hutson T.E., Kopyltsov E., Méndez-Vidal M.J. (2021). Lenvatinib plus Pembrolizumab or Everolimus for Advanced Renal Cell Carcinoma. N. Engl. J. Med..

[39] Shim M., Song C., Park S., Choi S.-K., Cho Y.M., Kim C.-S., Ahn H. (2015). Prognostic Significance of Platelet-Derived Growth Factor Receptor-β Expression in Localized Clear Cell Renal Cell Carcinoma. J. Cancer Res. Clin. Oncol..

[40] Song S.H., Jeong I.G., You D., Hong J.H., Hong B., Song C., Jung W.Y., Cho Y.M., Ahn H., Kim C.-S. (2014). VEGF/VEGFR2 and PDGF-B/PDGFR-β Expression in Non-Metastatic Renal Cell Carcinoma: A Retrospective Study in 1,091 Consecutive Patients. Int. J. Clin. Exp. Pathol..

[41] Vanharanta S., Shu W., Brenet F., Hakimi A.A., Heguy A., Viale A., Reuter V.E., Hsieh J.J.-D., Scandura J.M., Massagué J. (2013). Epigenetic Expansion of VHL-HIF Signal Output Drives Multi-Organ Metastasis in Renal Cancer. Nat. Med..

[42] Sanjana N.E., Shalem O., Zhang F. (2014). Improved Vectors and Genome-Wide Libraries for CRISPR Screening. Nat. Methods.

[43] Koike-Yusa H., Li Y., Tan E.-P., Velasco-Herrera M.D.C., Yusa K. (2014). Genome-Wide Recessive Genetic Screening in Mammalian Cells with a Lentiviral CRISPR-Guide RNA Library. Nat. Biotechnol..

