# Supplementary material for: Cancer Cell-Derived PDGFB Stimulates mTORC1 Activation in Renal Carcinoma
Source: Int J Mol Sci. 2023 Mar 29;24(7):6447. doi: 10.3390/ijms24076447 (PMC10095210; doi:10.3390/ijms24076447)
Supplement: Supplementary file 1 [file ijms-24-06447-s001.zip › ijms-2090539-supplementary.pdf]

Supplementary Table S1: sgRNA sequences, PCR and sequencing primer sequences

**Construct**	**Sequence (5′-3′)**
Top sgPDGFB_1	CACCGGTTGGAGATCATCAAAGGAGGT
Bottom sgPDGFB_1	TAAAACCTCCTTTGATGATCTCCAACC
Top sgPDGFB_2	CACCGCTGCTGCACGGAGACCCCGGGT
Bottom sgPDGFB_2	TAAAACCCGGGGTCTCCGTGCAGCAGC
U6 Promoter Fwd	GAGGGCCTATTTCCCATGATTCC
EcoRI-PDGFB CDS Fwd	ATCGGAATTCATGAATCGCTGCTGGGCGCT
XbaI-PDGFB CDS Rev	ATCGTCTAGACTAGGCTCCAAGGGTCTCCT
pCEP Fwd	AGAGCTCGTTTAGTGAACCG

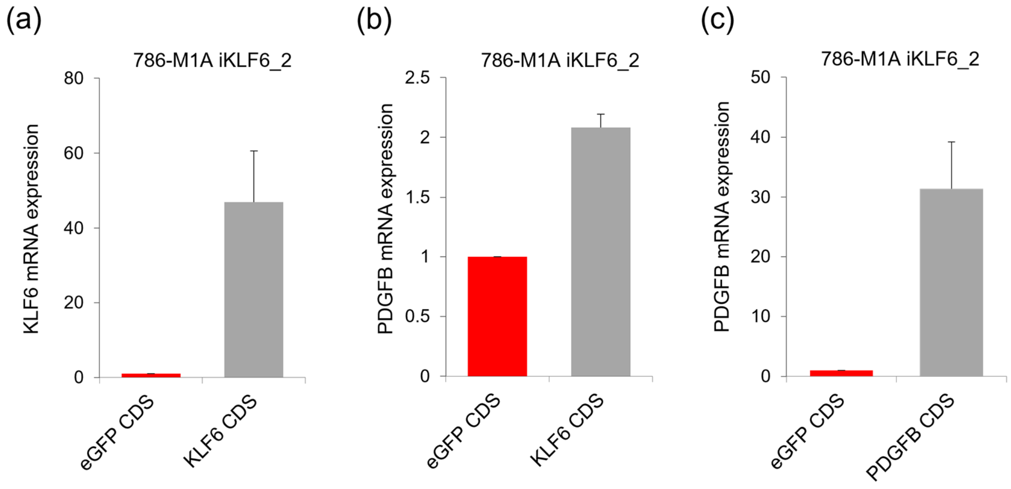

Supplementary Figure S1: (a-b) The expression of **(a)** KLF6 and **(b)** PDGFB upon the reintroduction of exogenous KLF6 into the KLF6-repressed 786-M1A cells. **(c)** The expression of PDGFB in the KLF6-repressed 786-M1A cells reintroduced with exogenous PDGFB. Average of three independent experiments. Error bars represent SD.

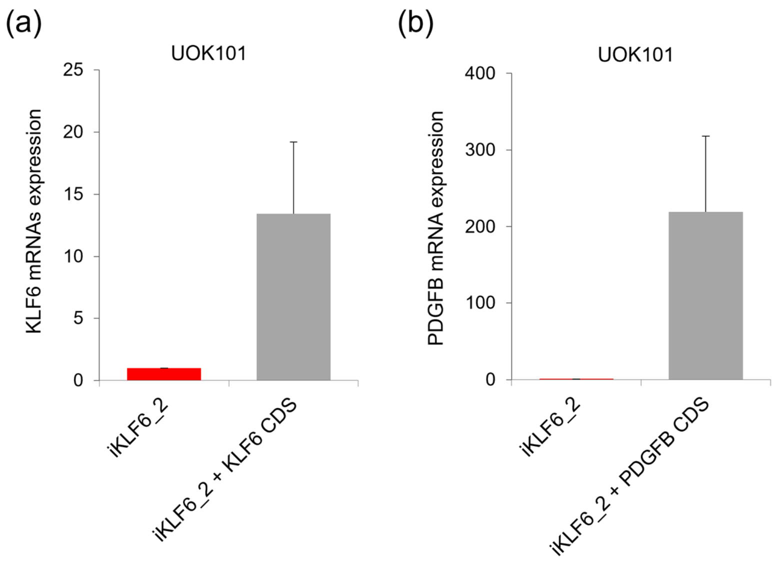

Supplementary Figure S2: (a-b) The expression of **(a)** KLF6 and **(b)** PDGFB upon the reintroduction of exogenous KLF6 CDS and PDGFB CDS into the KLF6-repressed UOK101 cells (UOK101 iKLF6_2), respectively. Average of two independent experiments. Error bars represent SD.

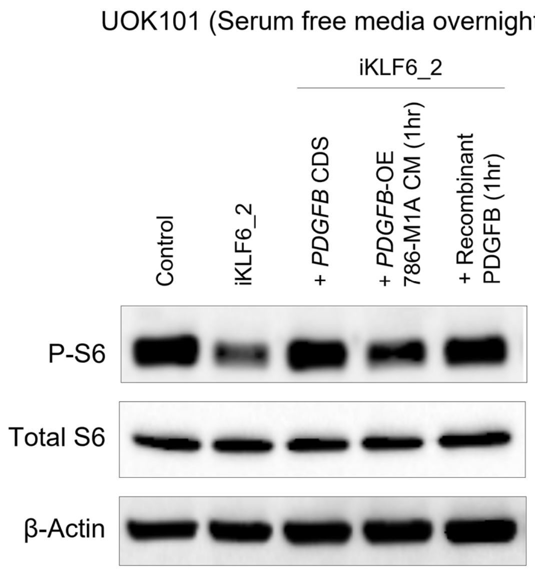

Supplementary Figure S3: mTORC1 activity, marked by ribosomal S6 phosphorylation level, of UOK101 control (1st lane), UOK101 iKLF6_2 (2nd lane), PDGFB CDS-expressing UOK101 iKLF6_2 (3rd lane), UOK101 iKLF6_2 that was cultured with PDGFB-overexpressing 786-M1A cells' conditioned media for 1 hour (4th lane) and UOK101 iKLF6_2 that was cultured with recombinant PDGFB for 1 hour (5th lane). The cells were cultured in serum free media overnight prior to the respective treatment and protein harvesting. Representative of two experiments
